# Nicotinamide riboside treatment enhances stress sensitivity and modulates hematological dynamics in aged mice

**DOI:** 10.1007/s11357-025-01793-5

**Published:** 2025-07-16

**Authors:** Luka Culig, Amogh Kashyap, Wakako Kuribayashi, Quia C. Claybourne, Isabel Beerman

**Affiliations:** 1 Epigenetics and Stem Cell Unit, Translational Gerontology Branch, National Institute on Aging, 251 Bayview Boulevard, Baltimore, MD 21224 USA; 2 Comparative Medicine Section, National Institute on Aging, National Institutes of Health, Baltimore, USA

**Keywords:** Nicotinamide riboside, Stress, Anxiety, Aging, NAD^+^

## Abstract

Nicotinamide riboside (NR) is a precursor to NAD^+^, a vital molecule for cellular energy metabolism whose levels decline with age. Aging is associated with loss of cognitive and immune function, alterations in hematological parameters, and increased vulnerability to stress. Although NR supplementation can mitigate age-related declines, it remains uncertain whether these positive effects persist when the organism is exposed to chronic physiological stress. We investigated this by exposing aged mice to NR supplementation alongside daily physiological stress and assessed various indices before and after treatment. Our results revealed that 6 weeks of NR supplementation protected against stress-induced thrombocytopenia and increased the frequencies of B and T cells. However, NR also heightened stress sensitivity, as evidenced by increased anxiety-like behaviors, while not affecting cognitive function. These findings suggest a dual role for NR in potentially enhancing immune function while exacerbating behavioral responses to stress. Future research on NR should consider stress as a variable to optimize its therapeutic usage in aging populations.

**Graphical Abstract:**

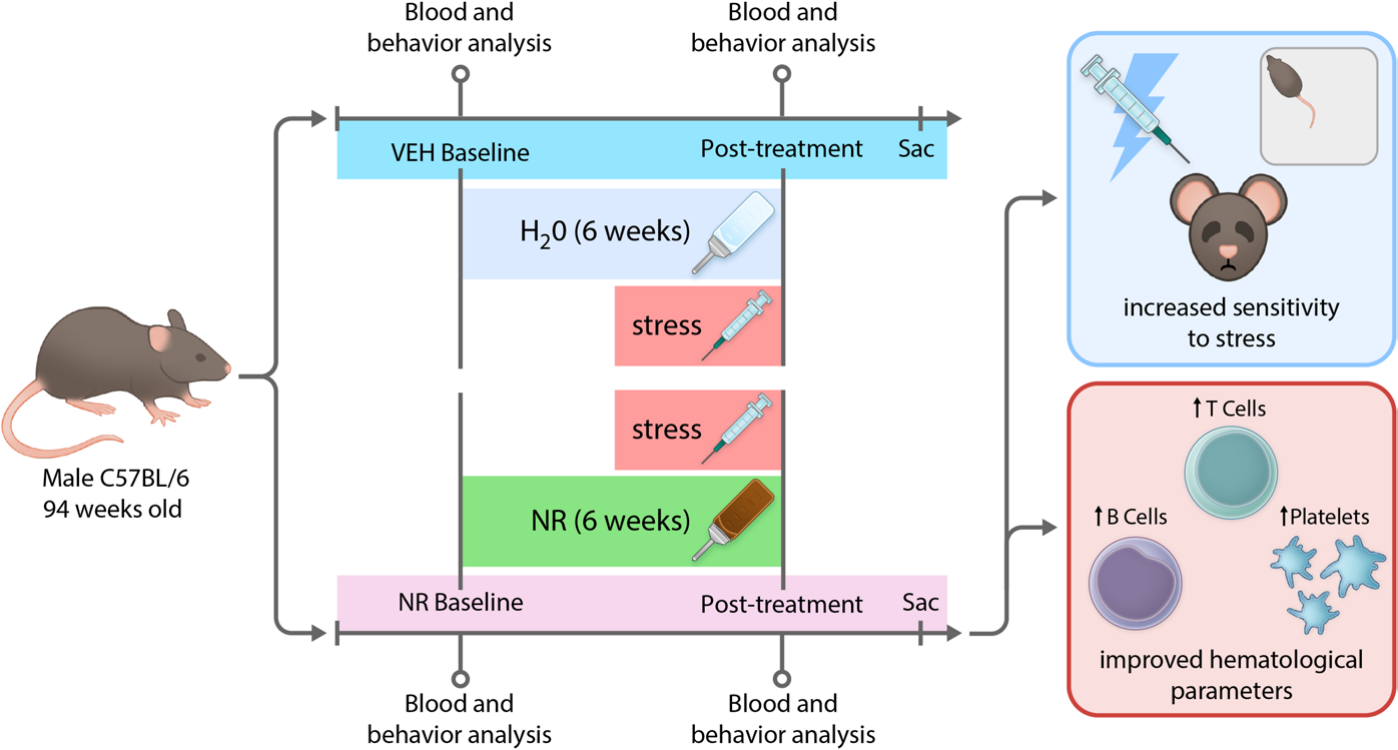

**Supplementary Information:**

The online version contains supplementary material available at 10.1007/s11357-025-01793-5.

## Introduction

Aging involves gradual, progressive deterioration that leads to loss of function and increased vulnerability to stressors that can result in death [1]. Stress, defined as maladaptive and nonspecific physiological dysregulation in response to environmental challenges [2], may accelerate aging [3], particularly later in life when physiological systems are already compromised [4]. Various types of stress impact the hematological and immune compartments [5]. For example, repeated cold stress and restraint plus water immersion stress both reduce the platelet count but increase the number of red blood cells (RBCs) in mice [6], while chronic unpredictable stress decreases RBC number without affecting platelets [7]. In humans, a mild psychological stressor [8] and aging [9, 10] increase natural killer (NK) cell levels. Mice exposed to 3 days of restraint stress display increased numbers of splenic NK cells, though longer-term restraint drives a loss of NK cells in the spleen [11]. Although splenic B-cell numbers do not seem to be affected by restraint, circulating B cells, another key component of adaptive immunity, also decrease in mice after exposure to restraint stress [12]. Furthermore, their number and diversity are compromised in old mice compared to young mice [13]. Aging itself is associated with a decreased B-cell generation rate, resulting from an increased hematopoietic stem cell (HSC) bias towards producing myeloid, rather than lymphoid cells [14]. The myeloid lineage is affected by stress and aging as well, with both repeated social defeat stress in mice and aging increasing the levels of neutrophils and monocytes (albeit in a sex-dependent way) [15, 16]. Lastly, T-cell function and repertoire decline with age in both mice and humans [17], while exposure to chronic stress increases their proportion in mouse blood [12]. We note the immune response to stress depends on several factors ranging from the intensity, chronicity, modality, and duration of the stressor, as well as the specific strain, sex, and age of the animal used, but highlight the sensitivity of this system to various forms of stress [18].

Exposure to stress has also been shown to affect lifespan and cognition. It has been argued that in both aging and stress, the neuroendocrine-immune communication via cytokines, hormones, and neurotransmitters is impaired and that chronic stress is linked with impaired health and premature aging in mice [19]. In fact, exposure to chronic social stress is able to shorten the lifespan of male mice [20], while high stress resistance is associated with longer lifespans in worms [21]. However, the modality, intensity, and duration of the stress all play a role here as well, evidenced by some studies showing a beneficial effect of mild stress on longevity [22]. The link to impairments in cognition has been suggested to occur through the age-dependent hypersecretion of the stress hormone corticosterone, which leads to hippocampal damage and further glucocorticoid dysregulation [23]. Many types of stress increase corticosterone in mice [24, 25], and increasing neurogenesis (which declines with age) can lower the elevated basal corticosterone levels in stressed animals [26]. Additionally, chronic stress also induces grey matter atrophy in rats [27] and alters functional brain networks in mice [28]. And just like stress can be an aggravating factor in aging, aging can also be an aggravating factor in stress sensitivity. For example, while mild repeated stress disrupts spatial working memory in both adult and aged mice, these negative cognitive consequences are exacerbated in aged mice [29]. Similarly, the physiological stress response has been found to be impaired in wild grey mouse lemurs, with aged animals exhibiting significantly higher stress hormone levels than younger animals [30]. Since both aging and stress drive impairments in cognition that can be rescued by exposure of aged animals to young blood [31], it has been suggested that putative lifespan-extending molecules and interventions could abate them [32], potentially through neurogenic effects in the hippocampal dentate gyrus, where newborn neurons have crucial roles in memory formation, learning, and resilience to and remission from stress [33].

One such molecule is nicotinamide riboside (NR), an NAD^+^ precursor vital for numerous enzymatic processes and cell metabolism whose levels decline with age [33]. Reduced NAD^+^ levels have been identified as an important causative factor in the pathogenesis of several DNA repair disorder-associated premature aging syndromes [34–36]. Consequently, its supplementation offers potential benefits (summarized in [37, 38]) including certain promising effects in humans [39]. In 16- to 18-month-old 3xTgAD/Polβ^+/−^ mice, a model of Alzheimer’s disease (AD), 3 months of NR supplementation improved learning, memory, and neurogenesis [40], and cognitive benefits occurred in another AD model (APP/PS1) as well as in wild-type 15-month-old (at NR onset) mice [41, 42]. However, the effects of NR supplementation on cognition in aged, stressed mice are unknown.

This study aimed to assess whether NR supplementation could ameliorate hematological and behavioral deficiencies in stressed, aged mice. We induced physiological stress via daily i.p. injections of a saline solution with 1% carboxymethyl cellulose and assessed the impacts of oral NR supplementation. Although NR demonstrated protective hematological effects, it also increased stress sensitivity in behavioral parameters, suggesting that NR in aging and stress should be a focus of continued studies.

## Results

### NR supplementation has a protective effect against stress-induced thrombocytopenia and blood alterations associated with enhanced immune function and reduced inflammation

**Fig. 1:**
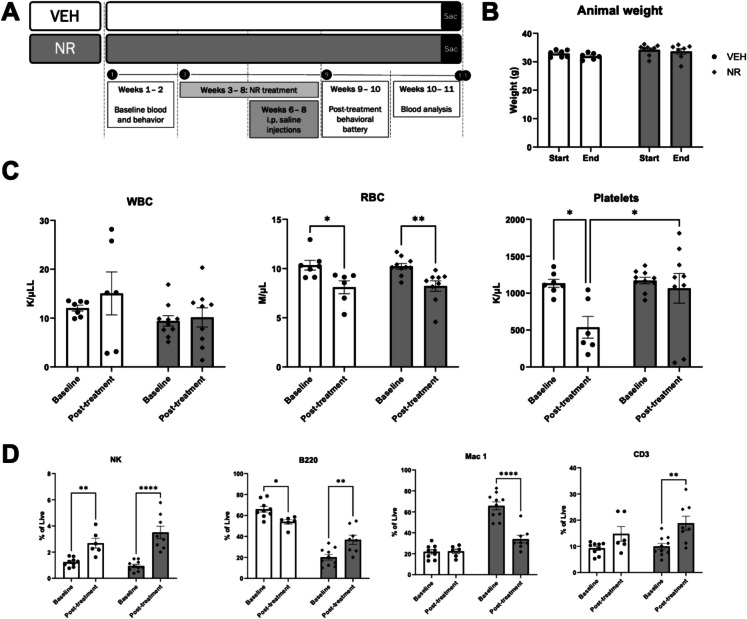
NR supplementation protects against a stress-induced decreases in platelets and B-cell frequency. **A** Schematic representation of experimental design. Mice were either exposed to 6 weeks of NR supplementation or maintained in standard conditions for the same duration. Both groups were treated with 21 daily intraperitoneal injections of a non-toxic vehicle during the last three weeks of NR supplementation. In weeks 9 and 10, mice were submitted to a battery of behavioral tests to assess cognition and stress-related behavior, while in weeks 10 and 11, the animals were sacrificed. **B** NR supplementation for 6 weeks did not significantly change the weight of mice. **C** Both groups demonstrated stress-induced decrease in RBCs, but the NR group maintained platelets after stress. **D** NK cell frequency was increased by stress in both conditions, and while B-cell frequency was decreased by stress in the VEH group, NR supplementation was able to increase B-cell frequency. In the VEH group, we did not observe changes in the frequency of myeloid cells, but NR supplementation decreased their frequency. Lastly, while we did not observe a significant effect on T cells in the control group, NR supplementation was able to induce an increase in their frequency. Data represent mean ± SEM. *p < 0.05; **p < 0.01; ****p < 0.0001. Abbreviations: VEH, vehicle; NR, nicotinamide riboside; WBC, white blood cells; RBC, red blood cells; NK, natural killer

Aged male C57BL/6 mice were divided into two groups: vehicle (VEH) and nicotinamide riboside (NR). In the first 2 weeks of the experiment, baseline measurements of peripheral blood and behavioral parameters were conducted to identify any pre-existing differences between the groups. At the start of week 3, NR supplementation was initiated in the NR group via drinking water and continued for 6 weeks (Fig. 1A). This duration was chosen based on previous studies demonstrating beneficial effects of 4–6 weeks of NR supplementation in aged mice HSCs [43]. Starting in week 6, 3 weeks after the initiation of NR supplementation, daily intraperitoneal (i.p.) injections were administered to both groups to induce physiological stress for 21 days. Previous research has identified multiple i.p. injections of a non-toxic vehicle (saline or cyclodextrin) as a form of chronic stress, associated with behavioral and physiological outcomes such as increased anxiety and elevated corticosterone levels [44, 45]. In weeks 9 and 10, following stress induction and NR supplementation, animals were assessed using a post-treatment behavioral battery. Between the end of week 10 and the beginning of week 11, after completing the behavioral assessments, the mice were sacrificed, and their blood was collected for analysis (Fig. 1A).

Neither stress nor NR supplementation had a significant effect on the body weight of mice, suggesting no adverse effects on weight regulation (Fig. 1B). To determine whether NR had a protective or buffering effect during stress on blood composition, we examined complete blood cell counts (CBC) in both groups at baseline and post-treatment timepoints. The results showed a stress-induced decrease in red blood cells (RBCs) in both groups, while platelets have significantly decreased numbers only in the vehicle group, suggesting a protective effect of NR against stress-induced thrombocytopenia (Fig. 1C). The increased variability in WBC counts post-treatment in the control group could be due to heterogeneity further increasing with age, implying that NR may help stabilize these counts. The two low platelet counts observed post-treatment in the NR group might be due to technical issues, and excluding these outliers would further support the protective role of NR.

Flow cytometry analysis of peripheral blood (Fig. 1D) revealed significant differences in lineage contribution across treatments. While the frequency of natural killer (NK) cells significantly increased in both groups (Fig. 1D), the effects on B cells, T cells, and myeloid cells varied across groups. Stress decreased the frequency of B cells in vehicle-treated mice, whereas NR treatment increased their frequency when compared to baseline measurement (Fig. 1D). Myeloid cell frequency was low and remained low after stress in the vehicle group, while in the NR group, their frequency was comparably elevated at baseline (due to mouse heterogeneity) but significantly decreased after stress after NR supplementation (Fig. 1D). We also recapitulated the previously reported effects of NR on the composition of Mac1^+^ cells of old mice [43]—specifically, a significant reduction in neutrophils and an increase in inflammatory monocytes (Supplementary Fig. 1). Lastly, the frequency of T-cell post-stress and post-treatment increased only in the NR group (Fig. 1D).

### NR supplementation may modulate behavioral changes towards a more stress-sensitive phenotype, without impacting cognitive functions

**Fig. 2:**
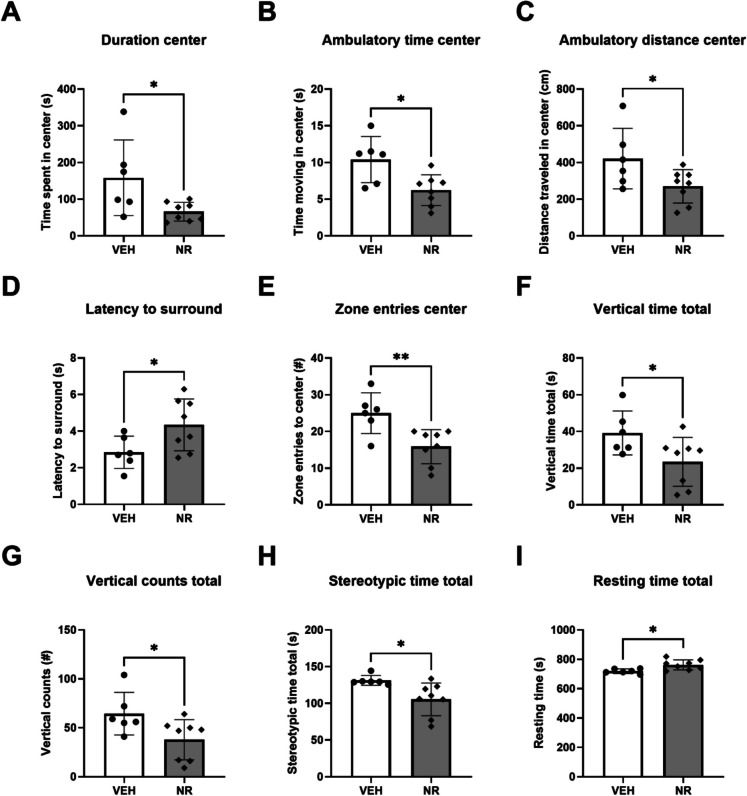
Post-stress post-treatment behavioral results from the open field test. Animals supplemented with NR spent less time overall (**A**), less time moving (**B**), and traveled less distance (**C**) in the center zone of the open field apparatus. They also spent less time rearing (**F**) and had fewer vertical counts across all zones (**G**). NR-treated animals took longer to enter the surround zone (**D**), and the number of zone entries to the center was decreased (**E**). NR-treated animals also had a reduction in stereotypic time spent (**H**) and an increase in resting time (**I**)

The open field test, carried out post-treatment, revealed multiple statistically significant changes between the groups, indicating an increase in anxiety-like behavior, a reduction in exploratory behavior, and a possible reduction in locomotor activity (Fig. 2). NR treatment significantly decreased overall time spent in the center zone, time spent moving in the center zone, and distance travelled in the center zone of the apparatus (Fig. 2A–C). Conversely, the NR group spent more time in the surround zone and had an increased latency to enter the surround zone of the apparatus (Fig. 2D). The number of entries to the center zone was significantly decreased in the NR-treated animals (Fig. 2E), which was accompanied by a reduction in the time and number of vertical counts (Fig. 2F–G). Additionally, NR decreased the stereotypic time and increased resting time (Fig. 2H–I). The open field results presented correspond exclusively to the post-treatment timepoint and not as a change from their baseline values. Although we assessed the animals in the open field test at baseline, this assessment was done in a different type of apparatus, preventing direct comparisons between the two timepoints. However, none of the parameters assessed showed significant differences between the treatment groups at baseline (Supplementary Fig. 2). Therefore, even with this potential caveat, it appears that NR supplementation may increase stress sensitivity, as evidenced by increased anxiety-like behavior and reduced exploratory activity in the open field test.

**Fig. 3:**
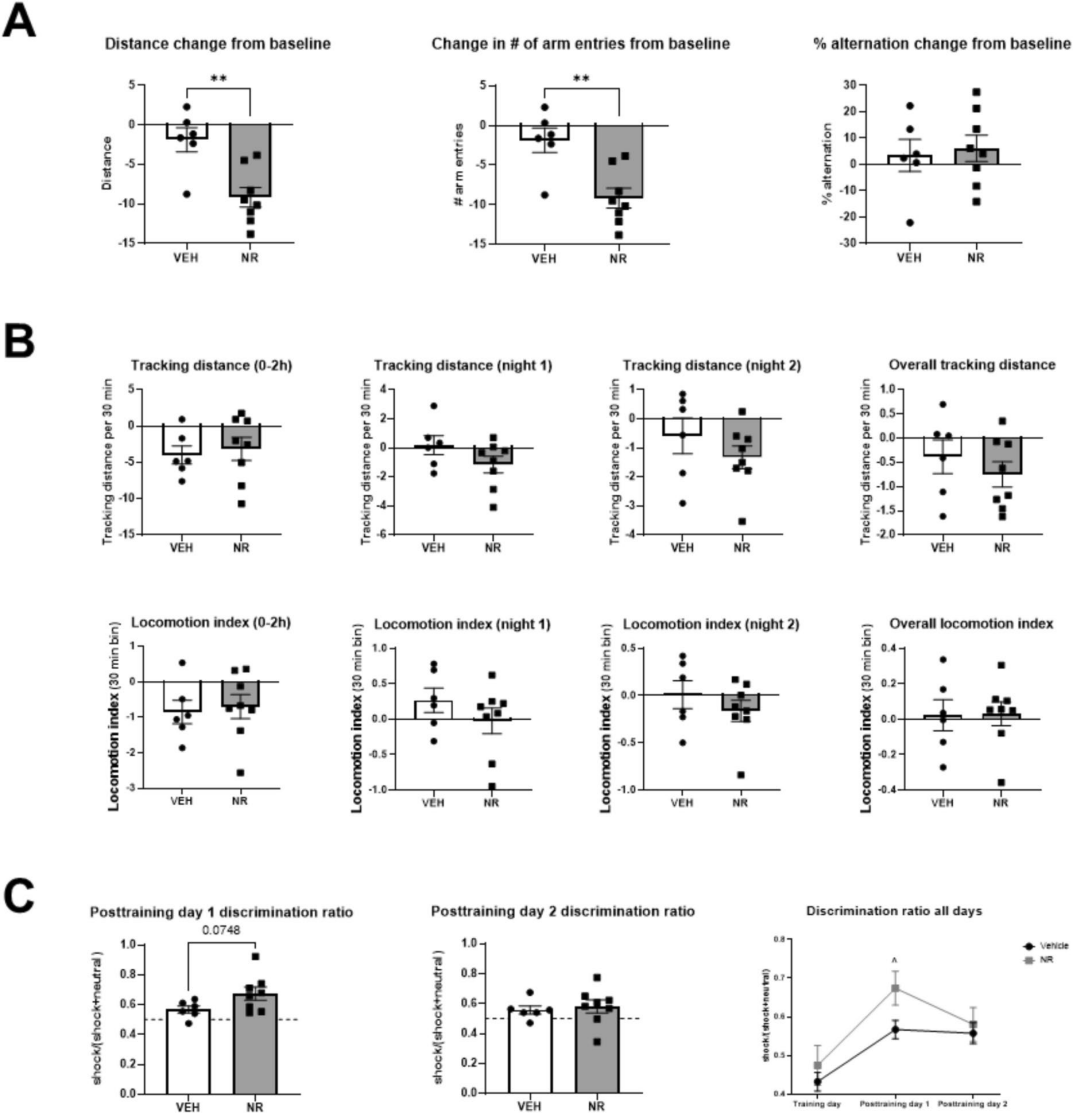
Behavioral changes from baseline to post-stress assessed by the cross-maze test, locomotor activity in the home cage, and fear context discrimination. **A** Compared to their baseline assessment, NR-treated animals moved significantly less and entered fewer arms of the four-arms cross maze apparatus, compared to the control group. No significant differences were observed in the % alternation compared to their respective baseline results. **B** No statistically significant differences between groups in changes of tracking distance or activity in the home cage. **C** No statistically significant differences in the ability of the animals to differentiate between the contexts in the fear context discrimination test. ^ denotes a trend of higher discrimination ratio in the NR-treated group on post-training day 1

In the four-arm cross maze test, we observed significant differences in the change of distance traveled and the number of arm entries, but no significant difference in the change of the %-alternation between the groups (Fig. 3A). While the control group showed no significant changes in these parameters from baseline to post-treatment, the NR group moved significantly less and entered fewer arms of the maze. There were no significant differences in any parameter from home cage activity testing (Fig. 3B) or in the fear context discrimination test. Neither the control nor NR-treated mice were able to robustly discriminate between the shock and neutral contexts, although there was a trend of a higher discrimination ratio between the contexts in the NR-treated animals on post-training day 1 (Fig. 3C). Taken together, these data suggest that NR treatment can lead to reduced exploratory behavior without affecting general locomotor activity, and it may also have subtle effects on context discrimination that warrant further investigation.

## Discussion

The NAD metabolome is dysregulated during aging and under various forms of stress in rodents [46] and humans [47], and it has been argued that NR, an NAD precursor, shows potential to support health during stress and aging in humans [48]. Given NR’s oral bioavailability and GRAS (generally regarded as safe) status, we examined its effects on old mice exposed to physiological stress. We focused on blood and neurological systems, supplementing mice with NR and exposing them to chronic stress (daily i.p. injections) during the last 3 weeks of supplementation to see if NR could buffer against the negative effects of stress [42, 43].

We determined that 3 weeks of daily injections were sufficient to induce stress, evident from a significant drop in RBCs and platelets in the control group, which is not typically seen with aging [43]. Future studies should include measurements of stress hormones like corticosterone to better evaluate stress levels. Despite stress and supplementation, body weight remained unaffected, indicating no toxicity or overt adverse effects.

In the hematopoietic compartment, we show that supplementing with NR prevents the reduction in platelet counts that is observed in vehicle-treated stressed mice, indicating its potential to buffer against stress-induced thrombocytopenia. However, NR was not able to protect against the stress-induced drop in RBCs, implying its differential impact on various cell types and highlighting the need for further research to understand the cellular pathways affected by NR in each system. This complex relationship between NAD^+^ precursors and RBCs is further evidenced by a human study where a different NAD^+^ precursor, niacin, decreased hemoglobin, and RBCs after 4 or 10 months of supplementation [49]. We found no significant changes in white blood cells (WBCs) after NR supplementation, which is also in line with results observed in the study cited above. While determining the mechanism behind this protective effect was beyond the scope of our study, we speculate that NR was able to protect against stress-induced thrombocytopenia by enhancing NAD^+^ levels, potentially improving mitochondrial function as reported in the primitive stem cell compartment [50] and downstream cells in addition to reducing oxidative stress. Indeed, NR can enhance HSC function by promoting mitochondrial clearance, leading to improved hematopoiesis in mice [51]. Furthermore, preclinical studies indicate that chronic psychological stress can induce alterations in various aspects of mitochondrial biology [52], while NAD + is known to support mitochondrial homeostasis, in part via the NAD + -mitophagy axis [53]. Although we did not directly assess mitochondrial parameters in this study, future investigations should address whether NR’s effects under stress involve modulation of mitochondrial function. We also did not measure NAD^+^ levels after supplementation; however, previous work from our lab with a similar 4–6 weeks 12 mM NR administration protocol leads to increased NAD^+^ levels in the bone marrow [54].

We observed a stress-related increase in the frequency of natural killer (NK) cells and a decrease in B-cell frequency. While the frequency of NK cells also increased after NR supplementation, B cells followed an opposite trajectory when compared to the control group, and their frequency was increased with NR. Additionally, NR supplementation led to alternative outcomes compared to vehicle, decreasing the frequencies of myeloid (Mac1^+^) cells, which were initially elevated in the NR group due to baseline heterogeneity. These opposing responses to stress with either vehicle or NR in these populations would not have been evident without baseline measurements, which also allow us to appreciate inherent variability in the aged cohort of animals.

Aging is typically associated with a decline in B-cell lymphopoiesis and a reduction in the diversity of the B-cell repertoire [55, 56], so the ability of NR to increase the B-cell frequency might indicate a rejuvenation or maintenance of the B-cell population, which may be beneficial for maintaining a robust adaptive immune response in aging. Similarly to the B-cell repertoire, aging is also associated with a decline in T-cell function and shrinkage of the T-cell repertoire due to a reduced T-cell potential of hematopoietic precursors and thymic involution [57]. These results support the potential of NR to increase the frequency of B-cells, contrasting the decline typically seen with aging [12] and stress [13], suggesting that NR may rejuvenate or maintain the B-cell repertoire, crucial for a robust adaptive immune response. The age-related decrease in B-cell generation arises from an increase in the bias of HSCs producing myeloid, rather than lymphoid cells [14], which points to a beneficial effect of NR on the proliferation capacity of HSCs in aged mice, avoiding the typical myeloid skewing. Further confirmation of this comes when looking specifically at the myeloid lineage, which drops precipitously after stress in the NR group, but not in the vehicle group. Lastly, the observed increase in T-cell frequency indicates a preservation or enhancement of T-cell mediated immunity, which is often compromised in aging [17]. Taken together, we hypothesize that NR enhanced HSC function and immune cell viability, which led to alterations in peripheral blood composition in the direction of rejuvenation. We have also reported on stress-induced changes in hematopoietic parameters that may be often overlooked when performing drug administrations through i.p. injections. Though NR is able to mitigate some of these stress-induced alterations, further research will be needed to determine the exact molecular pathways through which the effect on these immune cells is achieved.

Our behavioral analyses revealed that NR supplementation might increase stress sensitivity, shown by increased anxiety-like behavior and reduced exploratory activity in the open field test. Although baseline data points were collected using a different apparatus and are not directly comparable to post-treatment data, there were no significant differences at baseline. The observed post-treatment differences suggest that NR enhances stress sensitivity, indicating that pre-existing group differences are unlikely to have confounded the results. An increase in anxiety-related behavior (assessed by the elevated plus maze) has also been previously reported in APP/PS1 mice treated with NR, but not in aged mice on NR [42]. Therefore, we posit that NR increases stress sensitivity under perturbed conditions, such as chronic stress in aged mice, in the APP/PS1 model.

In conclusion, NR supplementation in aged, stressed mice protects against hematopoietic deficiencies, suggesting a potential rejuvenating effect on the immune system. However, NR also increased anxiety-like behavior, indicating that NR has a nuanced and context-dependent role. These findings underscore the importance of considering stress exposure when recommending NR supplementation, particularly in the elderly. Future studies should explore the molecular mechanisms behind NR’s dual effects to optimize its therapeutic benefits while mitigating adverse behavioral outcomes.

## Methods

### Animals

Male C57BL/6 mice aged 94 weeks (*n* = 16) at the beginning of the experiment were acquired from the NIA Aging colony. Animals were group-housed with littermates when possible, maintained on a 12-h light/dark cycle, and all testing was performed during the light cycle. All animal care and treatment were approved by the NIA Animal Care and Use Committee (ACUC), 469-TGB-2025, and were in accordance with the NIH Animal Guidelines.

### Experimental design

Animals were divided into two groups: vehicle (VEH, *n* = 7) and nicotinamide riboside treatment (NR, *n* = 9). At the start of the experiment, baseline measurements were taken from both groups and included blood sampling via retro-orbital bleeds for complete blood count, peripheral blood flow cytometry, and behavioral testing for locomotor activity in the home cage, open field, and cross maze testing. There was a 4-day gap between the retro-orbital bleed and placing the animals in the locomotor cages for assessing locomotor activity. Mice were initially assigned to groups randomly and then stratified based on weight and CBC parameters (RBC, WBC, platelet counts) to ensure no statistically significant differences between groups at baseline. On the following week, mice were habituated to handling for 3 days, and after habituation, open field and cross maze testing were done. In the third week of the experiment, the drinking water of NR animals was supplemented with 12 mM NR (Niagen®, Chromadex) for 6 weeks, while the vehicle group’s drinking water was not supplemented. Water bottles were changed two times per week and were freshly prepared each time. This duration of NR administration has been selected because of a previously reported increase in NAD with a similar (4–6 weeks) time frame [54]. Animals were intraperitoneally injected with 150–180 µL (based on body weight, ~ 0.05 mL/10 g) of a non-toxic vehicle 1% carboxymethylcellulose sodium salt (Sigma, C5678) solution in 0.9% saline (Baxter, 2B1324X), once daily for 3 weeks. To ensure sterility and minimize the risk of infection, the injection site was sprayed with 70% ethanol, and injections were administered using single-use insulin syringes with 28-gauge needles (BD, 329,424). Previous research has reported that repeated daily i.p. injections can elicit stress-related behavioral and biological phenotypes in mice [44, 45]. In weeks 9 and 10, animals underwent a post-stress post-treatment behavioral assessment consisting of locomotor activity tracking in the home cage, open field, cross maze, and fear context discrimination. At the end of week 10 and the start of week 11, animals were sacrificed, and blood was collected at euthanasia for complete blood count and flow cytometry analysis.

### Complete blood cell counts

Peripheral blood from mice was collected either retro-orbitally (at baseline) or via heart puncture (at sacrifice) into EDTA tubes (Sarstedt, 20.1278.100) that were placed on a rocker for 10 min prior to analysis on a Hemavet 950FS (Drew Scientific) according to the manufacturer’s instructions.

### Flow cytometry analysis

After complete blood cell counts, blood was treated with ACK buffer to lyse red blood cells, and stained with the following antibodies: FITC-conjugated anti-mouse Gr-1 (Biolegend, 108,406, 1:200), PE/Cyanine7-conjugated anti-mouse Mac1 (Biolegend, 101,216, 1:200), PerCP/Cyanine5.5-conjugated anti-mouse TER-119 (Biolegend, 116,228, 1:200), PE-conjugated anti-mouse CD3 (Biolegend, 100,206, 1:200), APC/Cyanine7 anti-mouse B220 (Biolegend, 103,224, 1:200), Pacific Blue™-conjugated anti-mouse CD4 (Biolegend, 100,428, 1:200), APC-conjugated anti-mouse CD8 (Biolegend, 100,712, 1:200), and Brilliant Violet 510™-conjugated anti-mouse NK-1.1 (Biolegend, 108,738, 1:200), on ice for 30 min. Immediately before running the samples, cells were resuspended in 140 µL of propidium iodide working solution to stain for viability. Analysis was performed using a BD FACS Canto II flow cytometer and Cytobank software (Beckman Coulter).

### Home-cage activity monitoring

Mice were temporarily switched from being group housed to being singly housed with activity automatically tracked using Digital Ventilated Cages (DVC, GM500, Tecniplast S.p.A., Buguggiate (VA), Italy) where activity was non-intrusively recorded by a capacitive-based sensor placed underneath each cage [58], 24/7 for the duration of 2 nights. Data acquired before 12:00 (first few 30 min bins) was discarded as a part of data cleanup, and the remaining data was analyzed in the following time bins: first 2 h in the cage after 12:00 pm, first night, second night, and overall data throughout the duration of the monitoring.

### Open field

To minimize the effect of repeated testing, we conducted the open field test in two different rooms, using two different systems. While this enabled us to minimize the carryover effects of subjecting the same set of animals to the same test, it introduces a challenge in comparing baseline to post-treatment data.

At baseline, open field was carried out by placing a mouse in a 40 cm × 40 cm × 40 cm white plexiglass chamber and recorded by overhead camera for 15 min. Behavior was analyzed with ANY-Maze software (Stoelting; Wood Dale, IL).

At post-treatment and post-stress, animals were placed in a 44 × 44 × 30 cm clear Plexiglas arena, located inside a dimly lit sound-attenuating cubicle. Activity was monitored for 15 min using an array of infrared beams and tracking software (MED-Associates; St. Albans, VT). Two zones were defined: the “center” zone (9 × 9 squares in the center of the apparatus, surrounded by 4 squares on each side) and the “surround” zone (everything else).

### Cross maze

Testing was performed as described previously [59]. Briefly, mice were placed in the center of an opaque red plexiglass maze containing four arms radiating from the center at 90° angles for 15 min. Arm entries and distance traveled were automatically detected using an overhead camera and ANY-Maze software. Alternations were scored as sequences of entries into three unique arms without repeats, and the alternation rate was calculated as the number of alternations divided by the number of opportunities.

### Fear context discrimination

Testing was based on a previously described method [60, 61]. Mice were assessed for their capability to differentiate between a shock-paired environment (context A) and a neutral environment (context B), which differ in floor texture, wall shape, white vs. infrared lighting, and odor. Housing rooms and method of transport to and from the fear conditioning room differed depending on the context used. The test consisted of three stages conducted consecutively: preexposure (1 days), training (1 day), and testing (2 days). During preexposure, mice were placed in shock-paired context A for 10 min in the morning and neutral context B in the afternoon for 10 min. Twenty-four hours later (Training; Day0), training began, and mice were put in context A for a total of 180 s. After a habituation period of 148 s (pre-shock period), mice received a 2 s, 0.5 mA electric foot shock. Test days (post-training days 1 and 2) follow an identical protocol to the training day (day 0) and only differ from day 0 by the fact that mice were exposed to shock in context A and had the opportunity to associate the two. Context A chamber featured straight 90° walls with stainless-steel grid rod flooring. White lighting in the box was on and 5 μl of mint extract (1:1 dilution with water, McCormick; Hunt Valley, MD) was placed in the collection tray of the box. Four hours later, the mice were placed in context B for 180 s without receiving a shock. Context B consisted of smooth plastic floor covering the metal rods and a triangle-shaped plastic insert above the inside of the chamber. White lighting in the box was turned off, and it was illuminated solely by near infrared lighting. Additionally, 5 μl of orange extract (1:1 dilution with water, McCormick) was applied to the ceiling of the chamber. Video Freeze software (Med Associates, St. Albans, Vermont) was used to measure and record freezing behavior exhibited by the mice during the testing sessions. All freezing scoring was done using default settings of the software. The analysis was made on the pre-shock time between context A and context B on test days. Discrimination ratio has been calculated as: (Time freezing in A)/(Time freezing in A + Time freezing in B). A score of 0.5 shows an inability to discriminate between the contexts, with freezing levels being the same in the shock and neutral contexts. A score of 1 indicates perfect discrimination, with animals freezing exclusively in the shock context and not at all in the neutral context. Similarly, a score of 0 also indicates perfect discrimination, but in the opposite direction: with animals freezing exclusively in the neutral context, but not at all in the shock-paired context. Therefore, after training, the higher the value above 0.5, the better the animal can discriminate, while the value of 0.5 represents chance value.

### Statistical analyses

All results are presented as mean ± SEM, and all statistical analyses were performed using GraphPad Prism 9. Due to missing values, body weight was analyzed by a mixed-effects two-way ANOVA without Geisser-Greenhouse correction, with timepoint and treatment as factors. Means comparisons for each timepoint (start vs. end) were conducted using a Bonferroni multiple comparisons test. Two-way ANOVA with Geisser-Greenhouse correction and post hoc multiple comparisons test (Bonferroni multiple comparisons test) was also used where appropriate. The difference between groups is considered statistically significant if *p* < 0.05.

## Supplementary Information

Below is the link to the electronic supplementary material.

ESM 1 (211 KB DOCX)

## Data Availability

All data is available upon request to Isabel.beerman@nih.gov.
