# Supplementary material for: Nicotinamide riboside treatment enhances stress sensitivity and modulates hematological dynamics in aged mice
Source: GeroScience. 2025 Jul 16;48(2):2451–64. doi: 10.1007/s11357-025-01793-5 (PMC12972475; doi:10.1007/s11357-025-01793-5)

**Supplementary Fig. 1. NR supplementation modulates the composition of Mac1+ cells from peripheral blood**

**The frequency of eosinophils was increased by stress in both conditions. NR supplementation decreased the frequency of neutrophils and increased the frequencies of both inflammatory and non-classical monocytes in the peripheral blood of old mice after stress.**


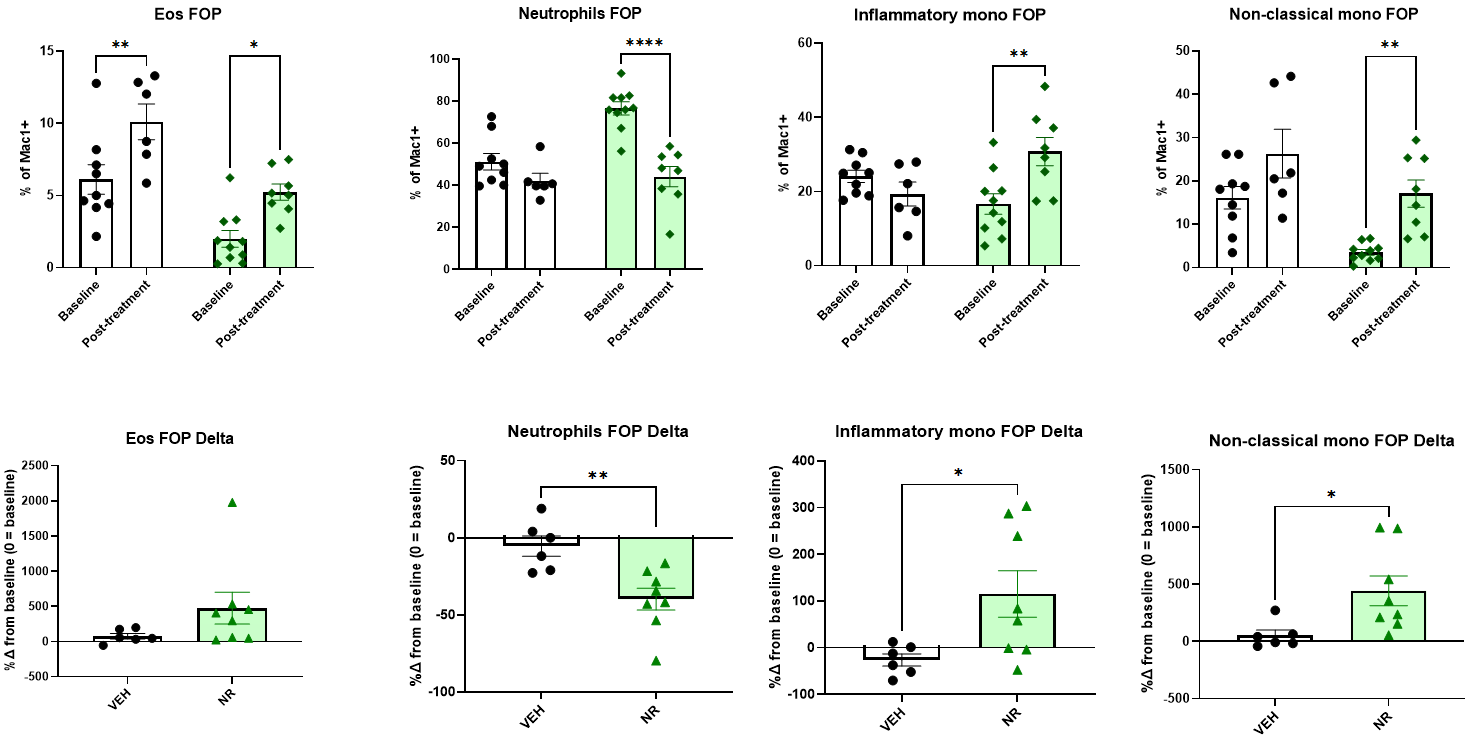


**Supplementary Fig. 2. Parameters in the open field test at baseline**

**All of the parameters assessed in the open field test at baseline showed no statistically significant differences between animals assigned to different groups.**


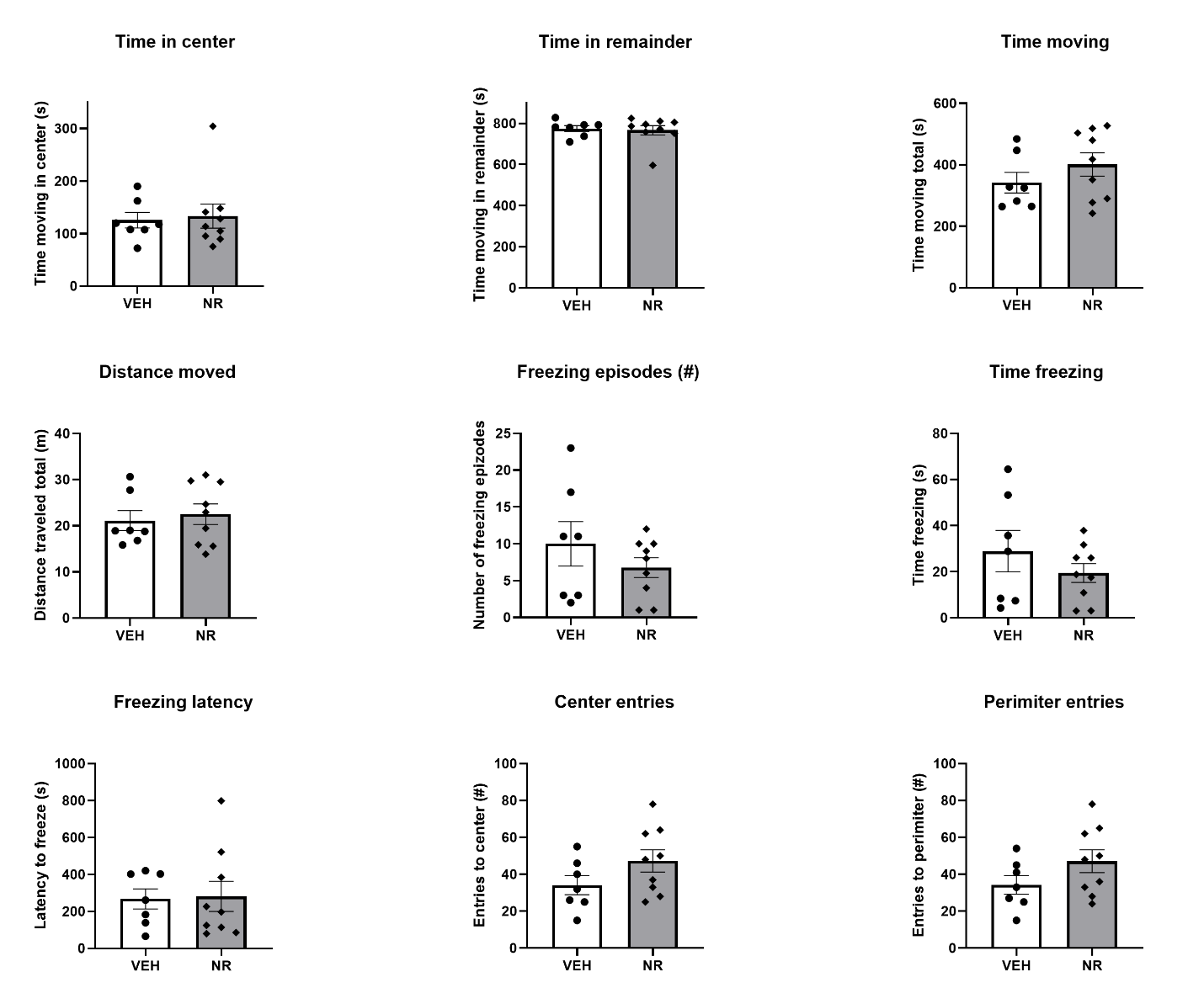

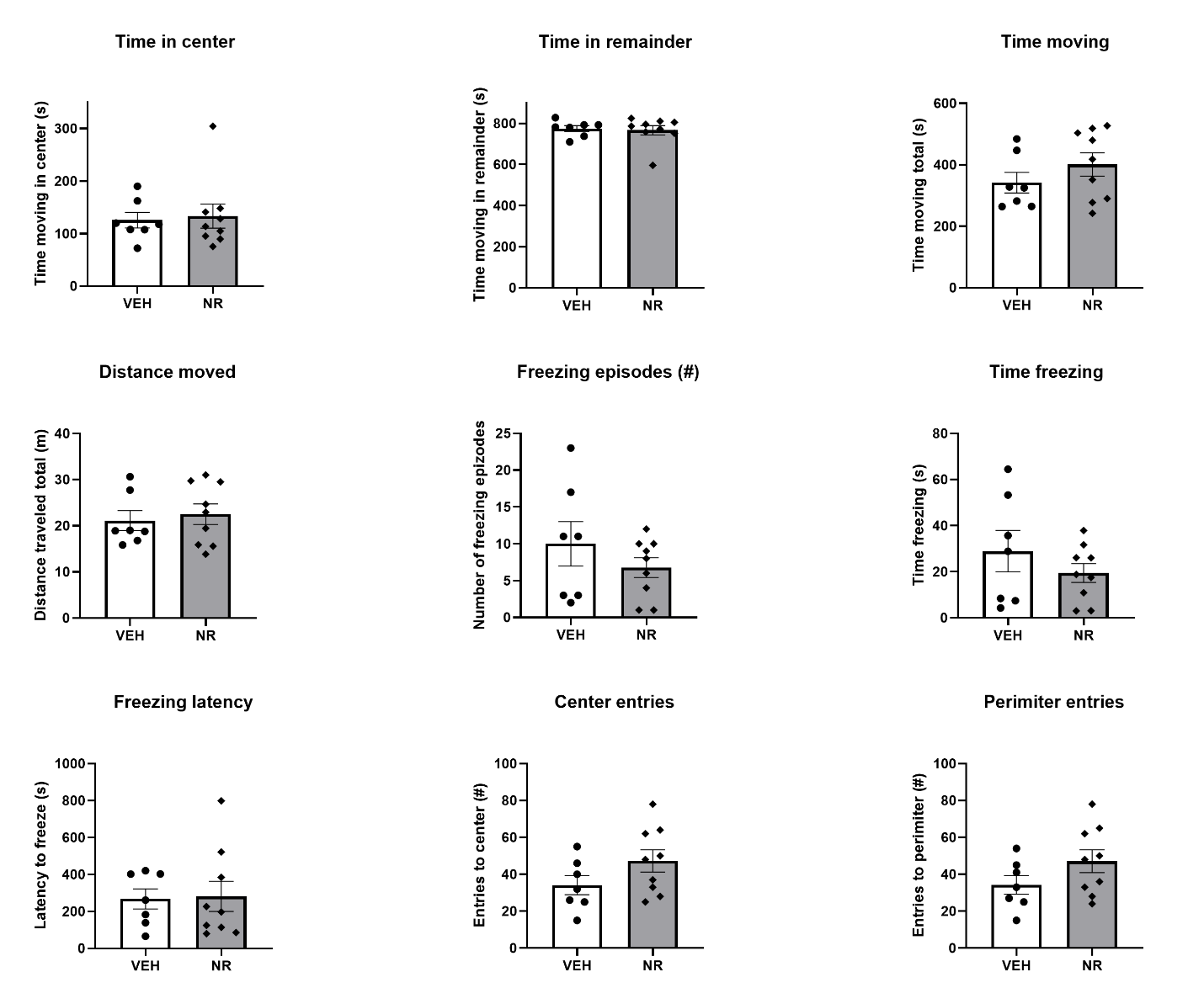

Supplement: Supplementary file 1 — (211 KB DOCX) [file 11357_2025_1793_MOESM1_ESM.docx]
